# Psychiatric Camouflage: A Case Series

**DOI:** 10.7759/cureus.72912

**Published:** 2024-11-03

**Authors:** Sreedevi S, Deepthi Pallurath Thekkethil, Bindu Menon

**Affiliations:** 1 Psychiatry, Amrita Institute of Medical Sciences and Research Center, Kochi, IND; 2 Psychiatry, Children and Young People's Services, Monkwearmouth Hospital, Sunderland, GBR

**Keywords:** frontotemporal dementia, nmda encephalitis, organic brain disorders, psychiatric manifestations, viral meningitis

## Abstract

Organic brain disorders are often camouflaged by psychiatric manifestations. Management of such ‘pseudo-psychiatric’ illnesses can be complicated due to the disruptive behaviour of the patients and/or lack of appropriate response to treatment. In this case series, we present three cases, each of which was initially diagnosed as a psychiatric illness but was later found to have an underlying neurological disorder. The presence of atypical symptoms, poor response to medications, disproportionate cognitive impairment and delirium should act as warning signs for the clinician to look for an underlying organic brain disorder.

## Introduction

The functional-organic distinction is a key concept in psychiatry and neurology that serves to differentiate between disorders with identifiable biological underpinnings and those without. Organic disorders are diagnosed when there is a high probability that examinations will reveal underlying cerebral or systemic issues contributing to the mental condition. In contrast, functional disorders lack sufficient identifiable pathophysiology to explain the symptoms. However, the boundary between organic and non-organic psychiatric disorders is often blurred, leading to ongoing uncertainty in many cases [1]. Organic brain disorders are often camouflaged by psychiatric manifestations. These “pseudopsychiatric emergencies” constitute 10% of all disorders in psychiatry ​[2]​. Evaluation and management of such patients can often be difficult and complicated due to their disruptive behaviour and/or lack of response to treatment [3]​. Any patient with an illness will have an emotional component attached to their condition due to their impairment and the associated suffering. These responses can be quite varied and depend upon factors such as the nature of the illness, its duration, the amount of impairment it results in, the social support the patients receive, and their personalities [4]. As clinicians, we must account for these factors to enhance our diagnostic accuracy, provide a tailored management plan, and improve patient outcomes. In this case series, we would like to call attention to the need for a detailed evaluation of patients presenting with psychiatric symptoms before classifying them as functional. These cases were selected from the psychiatry outpatient department of a tertiary hospital in Southern India. Informed consent was obtained from the patient/legal guardian to submit this case series.

## Case presentation

Case 1

**Figure 1:**
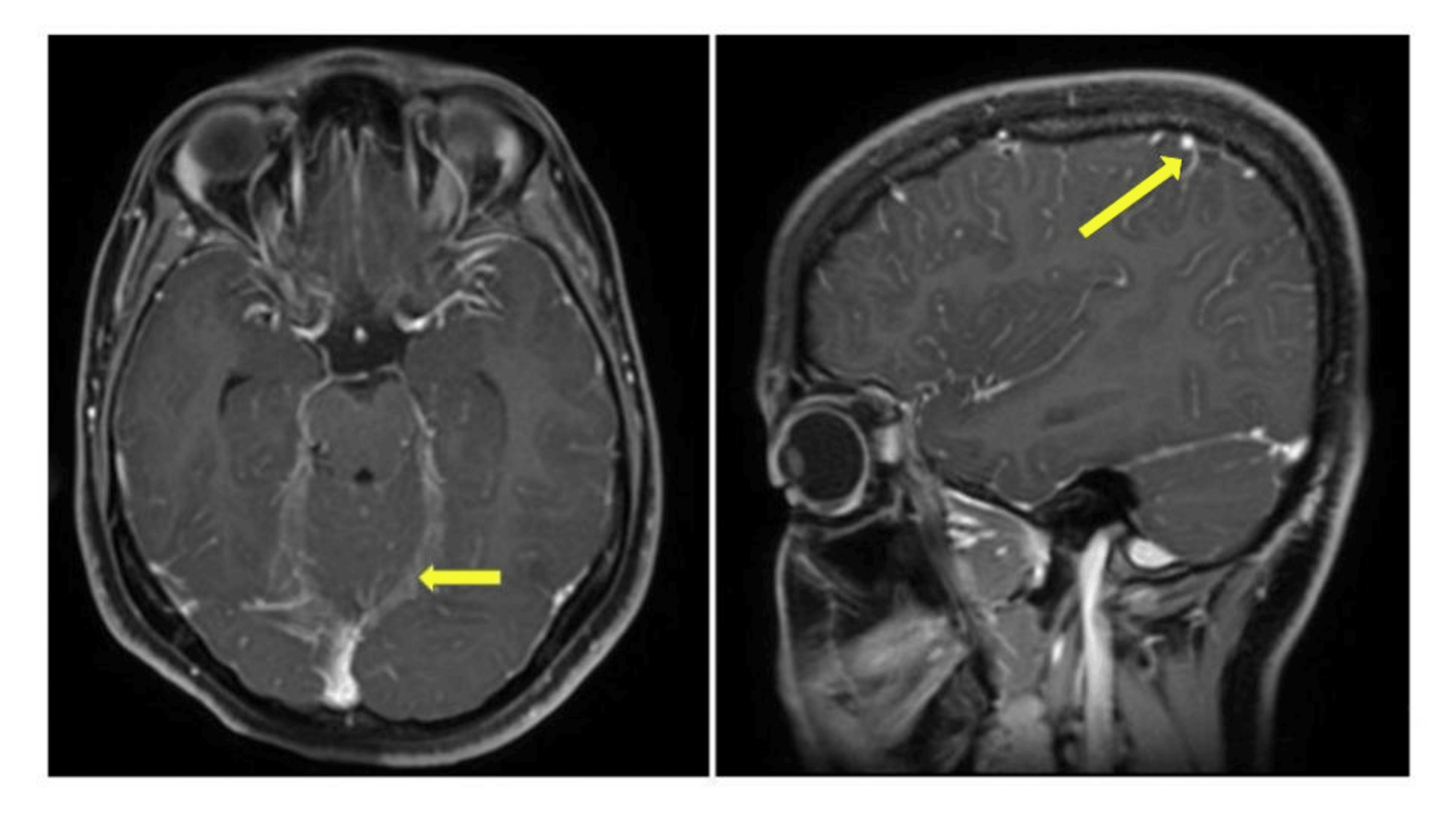
An MRI brain (axial and coronal view) showing subtle T2 FLAIR hyperintense signal intensities along the sulcal spaces of bilateral cerebral hemispheres with post-contrast mild diffuse pachymeningeal enhancement along the falx and tentorium; and leptomeningeal enhancement along sulcal spaces and cerebellar folia FLAIR: Fluid-Attenuated Inversion Recovery

A 20-year-old woman presented with a history of one episode of confusion and disjointed speech followed by persistent headaches and episodes of vomiting for 10 days. She had been taken to a hospital on the first day of onset of complaints where she was found to be oriented. She has not had any further episodes of confusion since then. She had contracted a COVID-19 infection a month ago, with symptoms such as fever, vomiting, headache and body pain. As the symptoms did not resolve, she was taken to the General Medicine department again where a computed tomography (CT) scan of the brain and blood tests were done. Apart from Vitamin D deficiency (8.94 ng/ml), she was not found to have any other abnormal reports. The patient’s parents had also reported stress secondary to her exams as she was not performing well in her academics for the past year. She was therefore referred to the psychiatry outpatient department (OPD) for further management considering the possibility of functional causes. Upon presentation, the patient displayed a marked reduction in response to environmental cues, including light, sound, and tactile stimuli, but could be aroused with repeated efforts. Additionally, she demonstrated a fluctuating loss of ability to move her limbs. Given these symptoms, she was admitted to the psychiatry department with a differential diagnosis of organic disorder versus mixed dissociative disorder (features suggestive of dissociative stupor and dissociative motor disorder) with psychogenic vomiting. Bilateral papilloedema was detected on the fundoscopic examination of the patient. Following this, a neurology consultation was undertaken, and she was advised magnetic resonance imaging (MRI) of the brain with magnetic resonance (MR) angiography and MR venogram (plain + contrast). Subtle T2 fluid-attenuated inversion recovery (FLAIR) hyperintense signal intensities were noted along the sulcal spaces of bilateral cerebral hemispheres with post-contrast mild diffuse pachymeningeal and leptomeningeal enhancement (Figure 1). 

Widening of the bilateral perioptic subarachnoid spaces with mild tortuosity of optic nerves was also found. The patient was then transferred to neurology in view of possibility of raised intracranial pressure. The cerebrospinal fluid (CSF) analysis showed a total count of 1692 cells/mm^3 ^and differential count showed all mononuclear cells with glucose levels of 78.7 mg/dL and protein levels of 145.1 mg/dL. The cytology was negative for malignant cells. The autoimmune panel and all the cultures in CSF were also negative. The patient was diagnosed with viral meningitis, and she was started on IV acyclovir and mannitol. She exhibited good clinical improvement and was discharged within a month. 

Case 2

**Figure 2:**
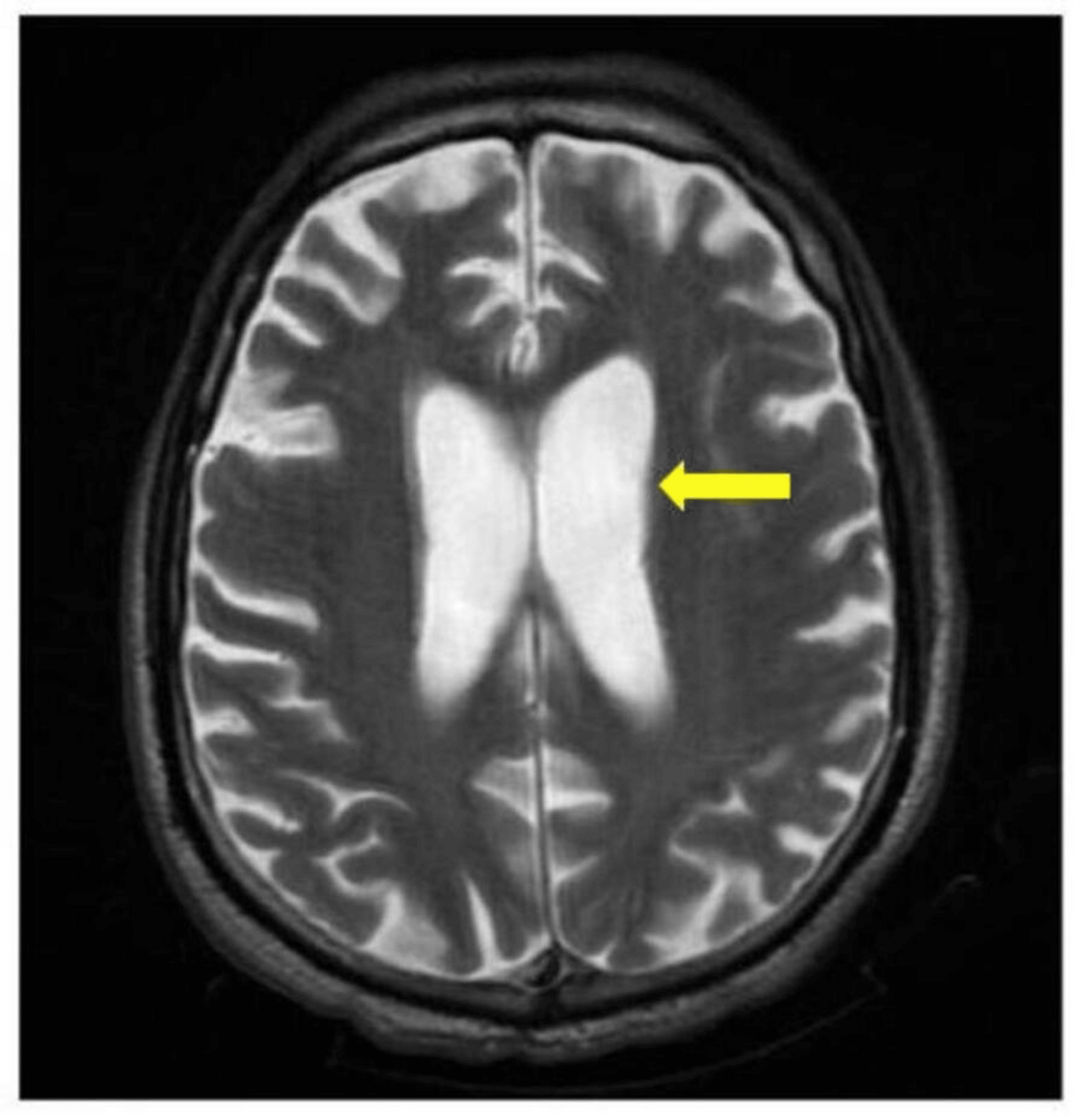
A plain MRI brain showing age-related neuroparenchymal changes with prominent sulcal spaces and ventricle

A 54-year-old man presented with a non-pervasive low mood, reduced interaction, crying spells, inability to do his work, difficulty counting and reduced sleep from four years following financial stressors. The patient's mood symptoms, such as crying spells and preoccupation with stressors, improved within two to three months following the resolution of stressors. However, he continued to experience low mood, reduced interaction, and cognitive impairments, including memory loss and an inability to manage finances. These deficits led to a referral to a neurologist, who prescribed donepezil 5 mg daily and vitamin B12 supplementation. MRI brain showed cerebral atrophic changes. The patient had worsening of non-pervasive affective and cognitive symptoms two years back following interpersonal stressors after which he was taken to a psychiatrist and started on escitalopram, fluvoxamine and methylphenidate (details regarding the doses were not known to the patient or his family). After two weeks of taking these medications, the patient developed overtalkativeness, increased sociability and excessive activity levels though he continued to have non-pervasive low mood and cognitive impairment. He was taken to another hospital where his medications were stopped, and all symptoms except low mood and cognitive impairment resolved. He was restarted on medications [escitalopram 30 mg and clonazepam 0.5 mg, both taken once daily (OD) at night)] and underwent 10 sessions of Transcranial Direct Current Stimulation (tDCS). He continued to take medications irregularly and received psychotherapy for a year after that. The patient was reported to be sad throughout this duration and have cognitive impairment. He had an insidious onset and gradual progression of extrapyramidal symptoms, including slowness of gait, tremors, and rigidity. Subsequently, all medications were discontinued, and the patient was initiated on trihexyphenidyl 2mg OD by an external practitioner to manage these symptoms. However, the patient’s symptoms worsened with onset of pill-rolling movements and marked reduction in speech. From two weeks prior to visiting our OPD, the patient developed refusal to eat food, irrelevant speech with fluctuating orientation, negativism and urinary as well as faecal incontinence. CT brain showed diffuse cerebral atrophy. The patient was again taken to another psychiatrist who started him on olanzapine 2.5 mg OD at night, escitalopram 5 mg twice daily (BD), trihexyphenidyl 2 mg OD, lorazepam 2 mg OD at night, Vitamin B12 supplementation, donepezil syrup 2.5 ml BD (2.5 mg BD) and L-Carnosine syrup 5 ml BD (100 mg BD). The patient was brought to our OPD as there was only slight improvement in oral intake with persistence of all other symptoms while on medications. There was no family history suggestive of parkinsonism or dementia. On examination, the patient had paratonia, bilateral rigidity, brisk deep tendon reflexes and frontal lobe release signs. He had stooped posture, short stepping gait and reduced arm swing. He scored 18 on the 23-item Bush Francis Catatonia Rating Scale with features of immobility, mutism, staring, rigidity, negativism, withdrawal, ambitendency and presence of grasp reflex. The lorazepam challenge test was given in view of catatonic signs, however there was no response. MRI brain showed significant neuroparenchymal atrophy (Figure 2).

A neurology referral was made and when the serum sample was tested, it was negative for the autoimmune encephalitis panel. Additionally, both thyroglobulin and thyroid peroxidase antibodies were within normal limits. Electroencephalogram (EEG) also did not show any significant abnormalities. The patient was diagnosed with probable Frontotemporal Dementia with Parkinsonism (FTDP) by the Department of Neurology based on findings of paratonia, apathy, anarthria, memory loss, difficulty handling finances, and a history of double incontinence, along with non-pervasive depressive symptoms. He was started on levodopa 100mg+carbidopa 25mg (half a tablet OD) and donepezil 5mg OD. He improved on treatment. He became continent and started communicating his need to urinate and defaecate. He started walking independently. The patient and his family members were informed about the diagnosis and advised to continue follow-up with the neurology department.

Case 3

**Figure 3:**
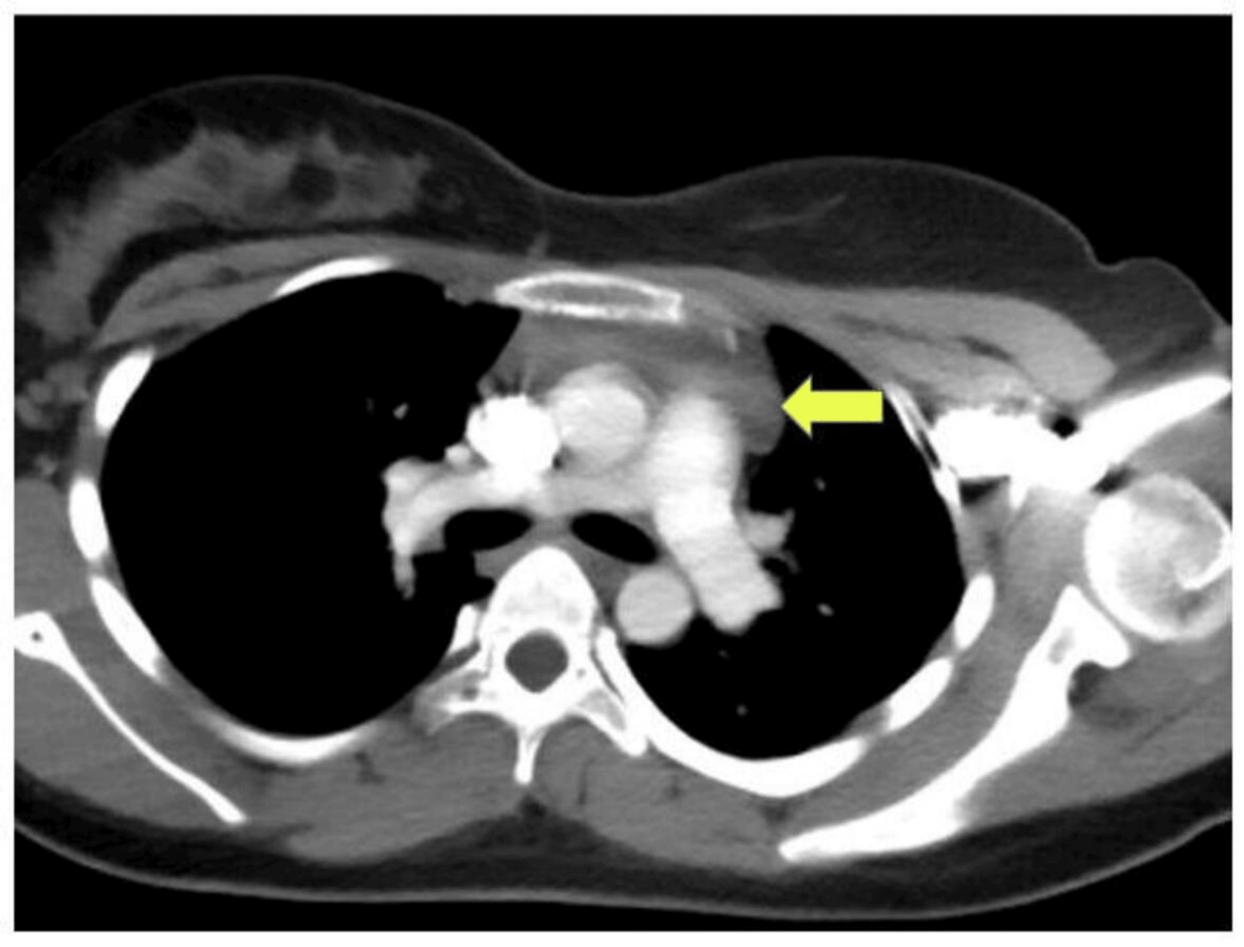
CT chest with contrast showing a bilobed mass with fat density and outer convex margins noted in the anterior mediastenum - thymic hyperplasia

A 14-year-old girl presented with a history of increased irritability, increased activity, irrelevant talk, neologisms, inappropriate laughter, disorganised behaviour, decreased sleep, and engaging in behaviours with potential for self-harm such as reaching to hold a moving fan from one month. The patient had a previous history of behavioural changes characterised by increased irritability, increased psychomotor activity, reduced sleep, abnormal posturing of hands, associated with fever and vomiting five years ago. She was suspected of having encephalitis and was treated with IV methylprednisolone, intravenous immunoglobulin (IVIG), and low dose risperidone. However, her anti-N-methyl-D-aspartate (NMDA) receptor antibody report was negative for serum and cerebrospinal fluid (CSF) samples at the time. Her CSF viral panels were also negative. The patient had a history of complaints similar to her current illness one year ago, with irritability, increased activity, irrelevant talk, inappropriate behaviour, and laughter, following an exam stressor, and had sought treatment from another hospital. Her symptoms had completely resolved with treatment lasting for two weeks with risperidone, fluoxetine and clonazepam. For her current illness, a differential diagnosis of acute polymorphic psychotic disorder versus mania with psychotic symptoms was made, and patient was started on olanzapine 5 mg OD (later increased to 5mg 1-0-3), valproic acid increased up to 250 mg (1-0-2) and chlorpromazine 50 mg OD. The patient had to be discharged against medical advice (DAMA) within a week as she contracted COVID-19, and parents opted to continue treatment at home rather than shift her to isolation. The patient was reviewed again after 10 days of isolation. She was readmitted and referred to paediatric neurology in view of a lack of response to medications. Her serum sample and CSF were very strongly positive for NMDA (Anti NR-1) type of glutamate receptor antibody. Prolonged eight-hour video EEG monitoring showed left frontotemporal dominant generalized non-specific disturbance of electrical function with excess of beta activity. Contrast-Enhanced Computed Tomography (CECT) chest showed a bilobed mass with fat density and outer convex margins in the anterior mediastinum, suggestive of thymic hyperplasia (Figure 3).

The patient was diagnosed with anti-NMDA receptor encephalitis based on a positive serum/CSF test for anti-NMDA receptor antibodies. Initial treatment with IV methylprednisolone followed by oral prednisolone in tapering doses resulted in minimal improvement. Valproic acid was stopped, and patient was continued on olanzapine 10 mg OD, chlorpromazine 50 mg BD and lorazepam 2 mg OD in view of severe agitation and aggression. Despite undergoing five cycles of plasmapheresis, there was no significant improvement in her condition. While the irrelevant talk and abnormal laughter decreased, the patient continued to exhibit increased irritability, anxiety, reduced sleep, agitation and disorganised behaviour. As the patient failed to improve on the two first-line therapies, two doses of inj. rituximab were given, and she was later started on mycophenolate mofetil. Significant clinical improvement was seen within 45 days and there was complete remission within two months. Psychotropic medications were also slowly tapered during this period. However, the patient was subsequently lost to follow-up.

## Discussion

The cases described above highlight the risk of psychiatric overshadowing, where organic brain disorders are misdiagnosed as purely psychiatric conditions. This misdiagnosis can lead to treatment delays, jeopardizing the chances of patients' remission and hindering their ability to lead productive lives. 

In the first case, the patient presented with a history suggestive of one episode of delirium followed by vomiting and headache. The patient was taken to a nearby hospital immediately and found to be oriented. She was referred to psychiatry to rule out functional causes of illness. However, conditions such as delirium may have a fluctuating course ​[5]​. These fluctuations may result in misdiagnosis and misattribution of signs to psychiatric disorders ​[6]​. Though the patient did not present with neuro-ophthalmological symptoms of illness, fundoscopy is still an integral part of neurological examinations and should be performed by any doctor, not simply restricted to ophthalmologists. It is the only method that facilitates direct visualization of the vasculature and the cerebrospinal nervous system in a living individual. In this case, the patient had symptoms that required assessment for elevated intracranial pressure ​[7]​. This case could have possibly been diagnosed earlier with a detailed medical history and a basic neurological examination, highlighting the need for a step-by-step approach, especially for patients with newly-diagnosed psychiatric disorders.

The behavioural variant of frontotemporal dementia (FTD) is well known for the exclusive or predominant prevalence of neuropsychiatric symptoms during the initial stages of the illness ​[8]​. The affected areas of the brain dictate the various psychiatric manifestations. Cases of Pick’s disease can be diagnosed inaccurately as depression due to the presence of symptoms such as amotivation, anhedonia, asociality, and apathetic attitude ​[9]​. Some patients with FTD, usually the behavioural variant, can develop symptoms of Parkinson’s disease. This is called FTDP ​[10,11]​. In the second case, the patient took four years to be diagnosed after the onset of symptoms. The patient and his family members ended up frequently changing practitioners and specialties due to a lack of clarity regarding the illness and appropriate treatment. Without a detailed chronological history and assessment, practitioners run the risk of diagnosing the patient inaccurately when they focus only on the cross-sectional presentation. Although a prolonged delay in diagnosis is often run-of-the-mill for FTD, early detection and diagnosis of FTD/FTDP are crucial for the effective planning of interventional strategies for these patients ​[12]​. Incorrect diagnosis can lead to delayed treatment, exacerbating the patient's and the family's distress. As a result, doctors should consider referring patients with mid-life or late-onset psychiatric symptoms for evaluation of neurodegenerative diseases ​[13]​. Once the diagnosis, prognosis, and treatment goals were clarified to the patient and the family members, they finally had a clear understanding of the required management plan. This helped improve the patient's outcome and mitigate caregiver distress.

In the third case, although the patient was treated for encephalitis at the age of nine, her autoimmune panel was negative, and she was also taking risperidone at the time. Given that she had experienced similar symptoms a year earlier, which resolved with antipsychotic and antidepressant treatment, there was no initial reason to suspect an underlying organic cause. The monitoring of her progress was further hindered by the patient contracting COVID-19 infection and subsequent DAMA. However, upon review, it became clear that the patient had a poor response to adequate doses of medication, prompting us to consider the possibility of an underlying organic cause. Even after initiating treatment, the lack of improvement raised concerns. In such cases, it is important to monitor the patient's progress and wait for improvement after a thorough evaluation and diagnosis. While it's essential to initiate treatment, prematurely adding excessive psychotropic medications can lead to adverse effects and drug interactions. Therefore, a careful balance is needed between initiating treatment and avoiding potential side effects. A slow taper of psychotropic medications can help prevent a rebound of psychiatric symptoms. The association between NMDA receptor antibodies and the emergence of new-onset psychiatric symptoms is well-documented ​[14]​. These disorders may initially present with psychiatric symptoms at the outset because of which there may be delay in diagnosis or misdiagnosis ​[15]​. If this had gone unchecked, this patient would have received psychotropic medications for an extended duration with poor response unless she started developing clear organic signs and symptoms. The lack of response to medications served as the only trigger for considering an organic cause in this case, highlighting the extent to which organic brain disorders can masquerade as psychiatric disorders. This emphasises the importance of regularly reevaluating the patient's condition, even if initial assessments do not suggest an organic aetiology.

Diagnosing psychiatric disorders, especially when there is a suspicion of underlying organic factors, requires a comprehensive approach. First, a thorough evaluation must be conducted, including a detailed patient history, a neurological examination, and a mental status examination. This should be accompanied by an evaluation of the relevant differential diagnoses to explore possible medical and psychiatric conditions that might be contributing to the patient's presentation. In cases where symptoms suggest potential organic pathology, it is essential to communicate and collaborate with the relevant department. This ensures that appropriate evaluations and interventions can be made, facilitating timely and accurate diagnosis, leading to effective treatment. Any deviation from this systematic approach can lead to unnecessary delays.

Atypical symptoms, poor response to medications, disproportionate cognitive impairment, and delirium should act as warning signs for the clinician to look for an underlying organic brain disorder ​[16]​. The diagnosis of a psychiatric disorder sets a precedent for various hindrances in the patient’s life such as a lag, disruption, or even cessation in seeking further medical treatment due to fear of stigma from society ​[17]​. Apprehension regarding the attitude of medical health professionals towards these patients deters them from seeking further help. Various studies have shown that patients with mental illness receive lower quality of care for their physical problems. Keeping these factors in mind, meticulous history taking and examination of the patient become even more crucial and serve as important diagnostic tools for improving patient outcomes. 

## Conclusions

The cases presented in this series highlight the importance of a comprehensive evaluation of patients presenting with psychiatric symptoms. Though the phenomenon of psychiatric overshadowing is well documented through the ages, patients continue to be at risk of misdiagnosis with functional disorders. Atypical symptoms, poor response to medications, disproportionate cognitive impairment, and delirium should act as red flags to look for underlying organicity. A psychiatric misdiagnosis can play havoc with a patient’s life and deter them from seeking further treatment. Meticulous medical history, mental status examination, and neurological assessment are crucial to prevent misdiagnosis while ensuring timely and effective treatment. Additionally, maintaining a high index of suspicion for organic brain disorders, even in patients with seemingly straightforward psychiatric presentations, can significantly improve patient outcomes. 
